# Predicting Peptide-Mediated Interactions on a Genome-Wide Scale

**DOI:** 10.1371/journal.pcbi.1004248

**Published:** 2015-05-04

**Authors:** T. Scott Chen, Donald Petrey, Jose Ignacio Garzon, Barry Honig

**Affiliations:** 1 Howard Hughes Medical Institute, Columbia University, New York, New York, United States of America; 2 Department of Systems Biology, Columbia University, New York, New York, United States of America; 3 Department of Biochemistry and Molecular Biophysics, Columbia University, New York, New York, United States of America; 4 Center for Computational Biology and Bioinformatics, Columbia University, New York, New York, United States of America; Tel Aviv University, ISRAEL

## Abstract

We describe a method to predict protein-protein interactions (PPIs) formed between structured domains and short peptide motifs. We take an integrative approach based on consensus patterns of known motifs in databases, structures of domain-motif complexes from the PDB and various sources of non-structural evidence. We combine this set of clues using a Bayesian classifier that reports the likelihood of an interaction and obtain significantly improved prediction performance when compared to individual sources of evidence and to previously reported algorithms. Our Bayesian approach was integrated into PrePPI, a structure-based PPI prediction method that, so far, has been limited to interactions formed between two structured domains. Around 80,000 new domain-motif mediated interactions were predicted, thus enhancing PrePPI’s coverage of the human protein interactome.

## Author Summary

Complexes formed between a structured domain on one protein and an unstructured peptide on another are ubiquitous. However, they are often quite difficult to detect experimentally. The development of computational approaches to predict domain-motif interactions is therefore an important goal. We report a method to predict domain-motif interactions using a Bayesian approach to integrate evidence from a variety of sources, including three-dimensional structural and non-structural information. The method was applied to the entire human proteome and showed significant improvement over existing methods. The method was incorporated into PrePPI, a computational pipeline for the prediction of protein-protein interactions that relies heavily on structural information. Approximately 80,000 new interactions were detected. The new PrePPI database provides easy access to about 400,000 human protein-protein interactions and should thus constitute a valuable resource in a variety of biological applications including the characterization of molecular interaction networks and, more generally, in the study of interactions mediated by proteins in families that may not be extensively studied experimentally.

## Introduction

Mapping the human protein interactome has important implications for understanding basic biology and human disease at the molecular level [1]. High-throughput (HT) experimental techniques such as yeast two-hybrid and tandem affinity purification have been developed and applied to discover protein-protein interactions (PPIs) in multiple organisms on a genome-wide scale [2]. However, these approaches have inherent limitations, leading to a substantial false positive rate [2, 3] with many interactions likely undiscovered due to high rates of false negatives [2, 4, 5]. The development of reliable computational approaches to identify PPIs is therefore an important alternative to HT experimental techniques [6, 7]. Computational predictions of PPIs are based on criteria such as sequence orthology [8], similarity in evolutionary history [9], genomic context [10], and literature curation [11]. Predictions based on detailed structural modeling of PPIs have also been developed [12] and recent approaches [13] that combine low resolution structural modeling with non-structural information have begun to expand the applicability of structure to a genome-wide scale. Interactions determined by HT experiments and computationally have been deposited in databases such as STRING [14] and PrePPI [13].

An important class of PPIs involves interactions between a short peptide motif of one partner, and a structured peptide recognition domain (PRD) from another [15–18]. Discoveries of new domain-motif interactions present unique challenges compared to domain-domain mediated interactions [16, 19]. For a few major PRD families such as PDZ and SH3 domains, HT experimental techniques [19–21] such as phage display have been used to derive binding preferences which can subsequently be used to scan a genome to identify proteins likely to bind a given PRD. Computational modeling has also been used to predict domain-motif interactions [22–27]. In these studies, models for domain-motif complexes are built and evaluated with either physical or statistics–based scoring functions. Despite much progress, the experimental or computational effort involved significantly limits the scope of these studies to a few PRD families so that methods that enable predictions for a larger number of PRD families are needed. Databases such as the eukaryotic linear motif resource [28] (ELM) provide consensus sequence patterns for peptide motifs binding to many different PRD families, and methods such as iELM have been developed to make new predictions based on such information [29]. However, these patterns are often derived from a limited amount of data (e.g. from a few known binding sequences), which necessarily limit their coverage and accuracy. Surveys of available structures of protein-peptide complexes in the PDB have also identified candidate binding motifs [30] as well as generic structural characteristics for binding interfaces [31], but overall structural information has not been widely used in predicting new interactions except for a few PRD families.

In this study we report a computational framework to predict interactions mediated by domain-motif interfaces. The method uses a Bayesian approach to integrate knowledge from the ELM database, domain-peptide structures from the PDB, and non-structural information. We have incorporated the method into PrePPI [13] and found that the addition of domain-motif predictions improves its performance in PPI detection. The new version of PrePPI now contains 400,000 PPI predictions.

## Results

### Predicting peptide-mediated PPIs

**Fig 1:**
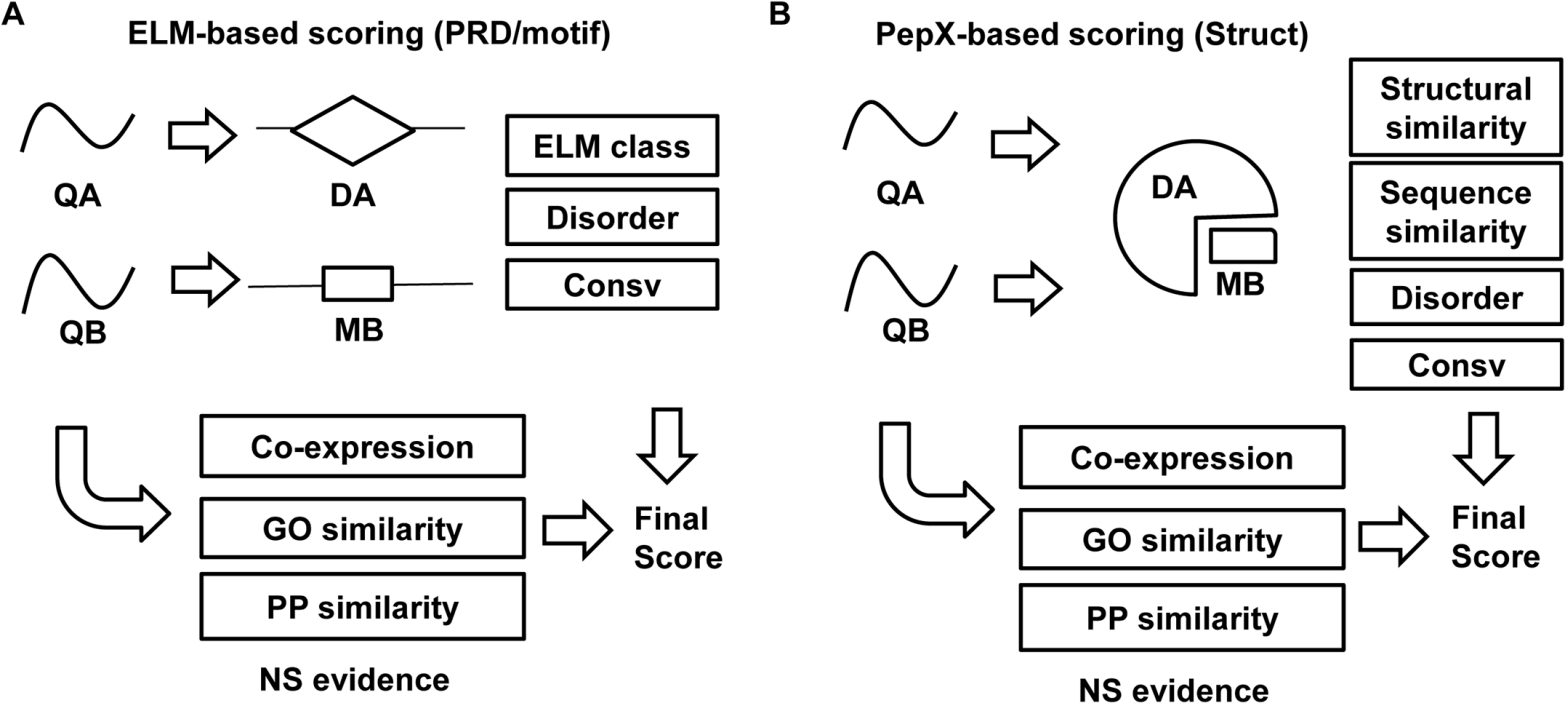
Predicting PPIs mediated by domain-motif interfaces. (A) Predictions made using information from ELM (method PRD/motif). For two query proteins QA and QB, if QA has a peptide recognition domain DA and QB has a motif MB from the same ELM class, a likelihood for a putative interaction between QA and QB was calculated (see Methods) based on the identity of the ELM class, predicted disorder of MB, and the sequence conservation of MB and combined with likelihoods from other non-structural (NS) evidence including gene co-expression, gene ontology (GO) similarity and phylogenetic profile (PP) similarity. (B) Predictions made using information from PepX (method Struct). For two query proteins QA and QB, a putative interaction between DA and MB is suggested using a template complex structure from PepX. A likelihood for the interaction is calculated based on the structural similarity between DA and the template PRD component, the sequence similarity between MB and the template peptide motif, disorder prediction, and sequence conservation of MB. Again this likelihood was combined with non-structural evidence to obtain a final score.

Fig 1 outlines the combination of strategies we use to predict PPIs mediated by domain-motif interfaces. The first approach we tried is based on ELM [28], a manually curated database containing more than 200 classes of PRD/motif pairs. In an ELM class a PRD is represented by its Pfam family and a motif is represented by a consensus sequence pattern derived from peptides known to bind to that family. For each ELM class, we identified all pairs of human proteins containing the corresponding PRD/motifs and calculated an interaction likelihood ratio (LR) for each. The LRs were calculated, using a Bayesian approach, as the percentage of pairs of proteins having the PRD/motif match in a true positive set of known human PPIs divided by the same percentage of a true negative set of 1.6 million pairs of proteins that do not interact (see Methods). In this calculation we also considered whether the motifs are located in predicted disordered regions and whether their sequences are conserved evolutionarily (see Methods for details). Sequence conservation and disorder have been shown to be associated with functional motifs [32, 33].

**Fig 2:**
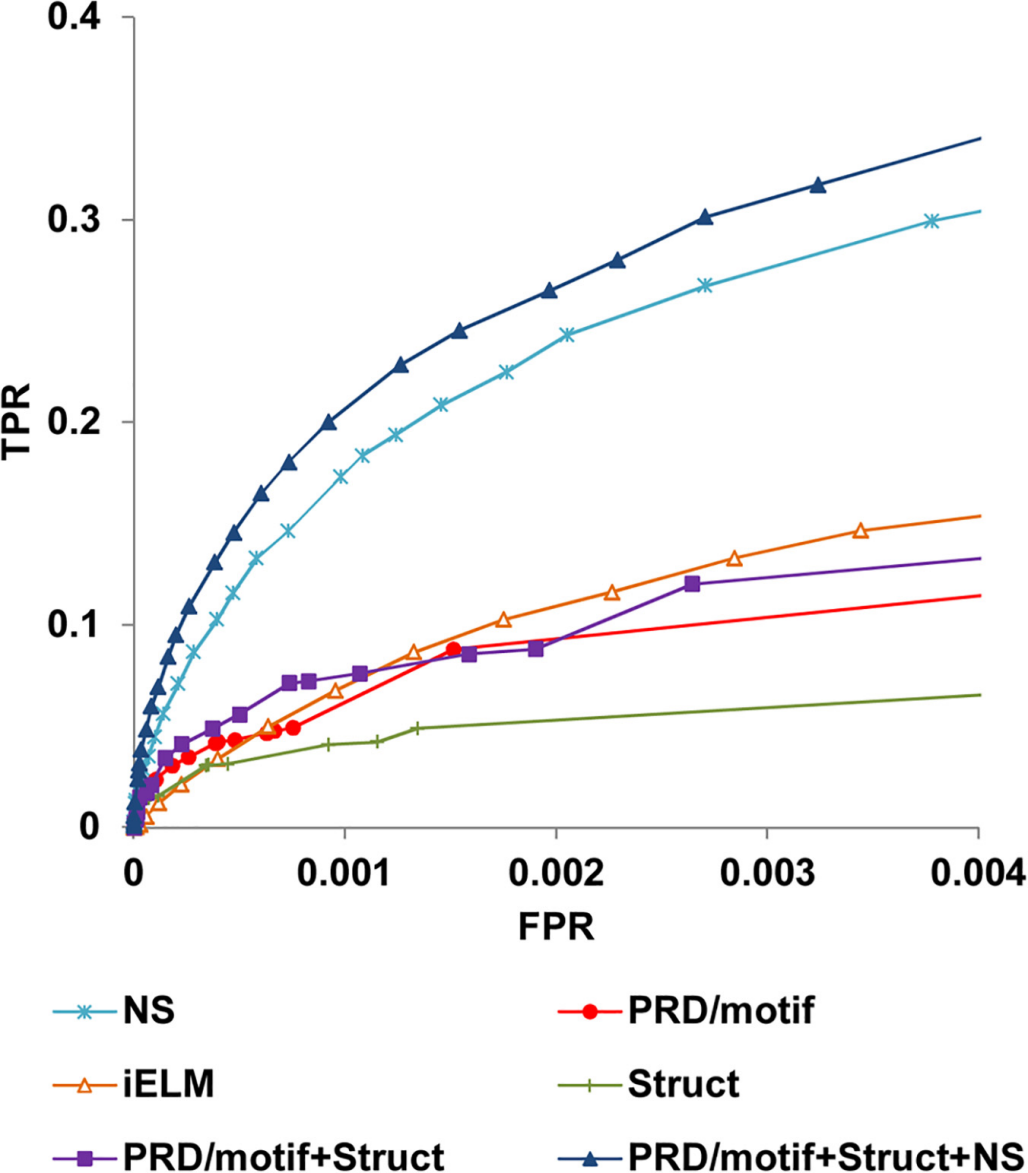
Prediction performance using different sources of evidence. True positive rates (TPR) versus false positive rates (FPR) in rediscovering human PPIs.

The performance of the PRD/motif predictor in rediscovering known human-human interactions was assessed using 5-fold cross-validation on the true positive and negative sets (see Methods) and compared with the iELM method developed by Weatheritt et al. [29] (Fig 2). iELM is also based on information from the ELM database and uses features such as sequence conservation and disorder incorporated into a support vector machine (SVM). In addition, instead of using Pfam to identify PRDs, Weatheritt et al. constructed their own Hidden Markov Models (HMMs) for each ELM class. As can be seen in Fig 2, PRD/motif performs better than iELM in the lower false positive region but the reverse is true in the higher false positive region. Similar results were obtained when using a precision-recall curve to evaluate performances, with PRD/motif having higher precision in the lower but not the higher recall region (S1 Fig). In what follows, we chose to use PRD/motif when extracting information from the ELM database based on its better performance in the lower false positive region.

Despite its broad scope, certain domain-motif interactions, especially those not belonging to well-studied families, may not be included in the ELM database. To expand our coverage, we used experimentally determined complexes taken from the PepX database [34] as templates to model domain-motif interactions (Fig 1). PepX contains high-resolution structures of protein-peptide complexes in the PDB whose peptide motif length ranges from 5 to 35 amino acids. Structural models for individual human proteins or their subdomains were retrieved from the PDB if available or from one of two homology model databases, ModBase [35] and SkyBase [36]. More than 10,000 human proteins have at least some part of their sequences covered by a structural model [13]. An interaction model for a pair of proteins was constructed if one protein contained a PRD that was structurally similar to a PRD in a given template in PepX and the other protein contained a short sequence motif with sequence similarity (based on BLOSUM62 scores [37], see Methods) to the motif component of the template. We only considered motifs whose BLOSUM scores ranked among the top 0.05% from the entire human proteome to retain a manageable number of candidate peptide motifs.

We again used a Bayesian approach to estimate the likelihood of an interaction given the properties of the model. Sources of evidence integrated into our Bayesian scheme include the sequence similarity score between the candidate motif and the motif in the template, the structural similarity score between the candidate PRD and the PRD in the template, whether the candidate motif is located in predicted disordered regions, and whether sequences around the candidate motif are conserved evolutionarily (see Methods). The resulting predictor, referred to as Struct for Structural information alone (based on the PepX database), performed worse than PRD/motif in our cross validation test. However, a predictor that combines both (PRD/motif+Struct) performs better than PRD/motif alone, showing that structural evidence is adding value to the predictions (Fig 2).

We combined PRD/motif-based and Struct-based LRs with non-structural (NS) evidence that has previously been used to infer PPIs [38]. Specifically, for each pair of proteins, we considered their co-expression level, their gene ontology (GO) functional similarity, and their phylogenetic profile similarity. Derivation of LR scores for these sources of evidence was described previously [13, 38] and the values obtained in our previous study [13] were directly used in the current one. A final score was obtained by multiplying the LR for the predicted domain-motif interface with the LR from non-structural evidence. The resulting integrative predictor, PRD/motif+Struct+NS, was then compared to the method based only on NS evidence in rediscovering known human PPIs (Fig 2). The NS-based method outperforms PRD/motif and Struct, which is not surprising given that NS is not limited to peptide-mediated interactions. However, PRD/motif+Struct+NS offers a significant improvement over NS alone (Fig 2). Furthermore, the combination of methods dramatically increases the number of predicted interactions with LR score > 600 [13, 38], referred to as “strong predictions” in this study. This LR value corresponds to a posterior probability of 0.5 that two proteins interact when assuming a prior odds of 1 in every 600 protein pairs interact. Details of the derivation can be found in Jansen et al. [38]. Using information from PRD/motif, Struct or NS alone led to 1,515; 0; and 15,376 strong predictions, respectively. In contrast, a total of 125,624 predictions were made when combining the three sources of evidence, representing 110,248 new predictions as compared to NS alone. This significant amplification highlights the value of combining independent clues. Notably, a total of 55 true positives can be detected before encountering the first false positive.

To obtain further validation of our approach, we compared our strong predictions to the 257 known human domain-motif interactions found in the ELM database. A total of 75 known interactions were included in our strong predictions (123 when using a LR cutoff of 100), while using only evidence from PRD/motif, Struct or NS alone recovered only 6; 0; and 6 interactions, respectively. Furthermore, when using the combined sources of evidence, the LR scores for more than half of the interactions (40 out of 75) ranked among the top 20% of all strong predictions. These 75 interactions were not dominated by a particular ELM class as they spanned 33 out of the 57 classes that represent the 257 known human interactions. We also examined overlap of our predictions with 160 human domain-motif complexes in PepX. The overlap is only 42 for strong predictions but increases to 86 when using a lower LR cutoff at 100.

### Incorporating peptide-mediated PPI prediction methods into PrePPI

**Fig 3:**
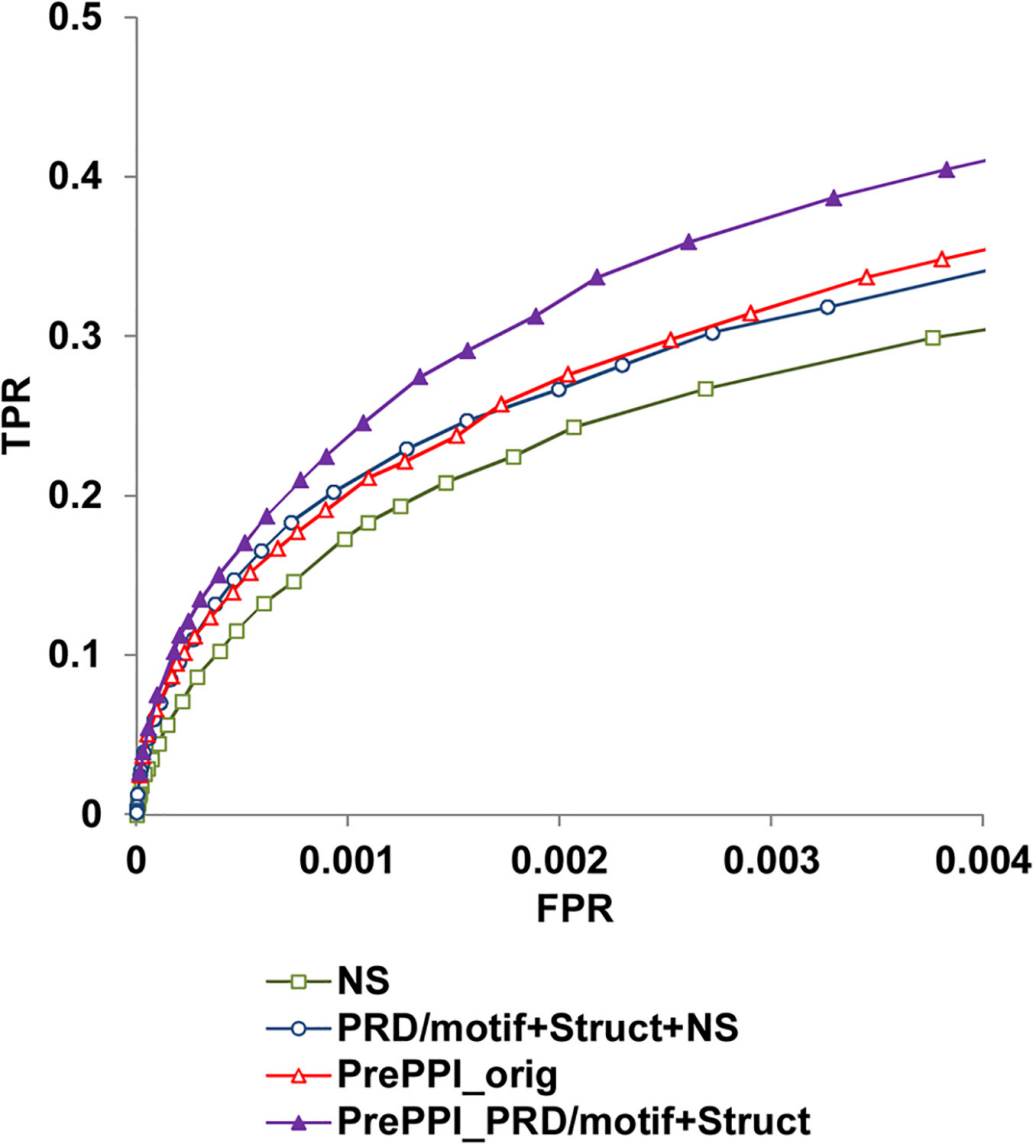
Improving PrePPI by adding domain-motif prediction methods. Prediction performance for PrePPI_PRD/motif+Struct compared to PrePPI_orig, PRD/motif+Struct+NS and NS only.

As summarized above, we have previously developed PrePPI, a computational PPI prediction method that performs comparably to experimental HT approaches. PrePPI combines structural evidence with non-structural evidence using a Bayesian framework but currently lacks the capability to predict domain-motif mediated PPIs [13], To add this ability we compared the structure-based LR from the original PrePPI and the LR for domain-motif interfaces obtained from PRD/motif+Struct (see Methods). The larger of the two was chosen and multiplied with the LR obtained from NS evidence to generate a final LR for the interaction. As shown in Fig 3, the addition of evidence based on domain-motif interactions (PrePPI_PRD/motif+Struct) results in improvement in performance when compared to the original PrePPI (PrePPI_orig). In this comparison, we use the same true positive set described above but a larger true negative set of non-interacting pairs of proteins which was used in the original PrePPI (performance is nearly identical for both true negative sets, S2 Fig).

PrePPI_PRD/motif+Struct yielded 78,898 additional strong predictions compared to PrePPI_orig. Although more than 40% of the predictions come from the 5 most prevalent PRD families (including SH2, SH3, 14-3-3, nuclear receptors and AGC kinase docking motif), over 130 ELM classes and 150 clusters of PepX template structures contributed to our results. Together with the original 317,814 interactions reported from PrePPI_orig, the new PrePPI which includes domain-motif mediated interactions contains a total of 396,712 predicted human PPIs.

## Discussion

In this study we developed methods to predict PPIs mediated by domain-motif interfaces using both expert knowledge of domain-motif interactions in the ELM database and structures of domain-motif complexes in the PDB. Although there is some overlap between predictions made with the ELM and the structure-based approach (PRD/motif and Struct), differences between them likely led to the observed improvements when two strategies were combined. For example, the Bcl-2 families have multiple structural representatives in the PDB but are not included in ELM. Moreover, the sequence similarity scoring approach of PrePPI_Struct allows the identification of motifs outside of the consensus provided by ELM. For example, the motif sequences for several SH3 and nuclear box receptor complexes in PepX could not be described by consensus patterns from any of their corresponding classes in ELM. Overall, among our new strong predictions, 13,988 are made using motifs that cannot be described by consensus patterns from the corresponding ELM class. On the other hand, the use of consensus patterns as in ELM (and hence PrePPI_PRD/motif) can be effective in capturing the variability of motifs for large, well-studied families even when no structural information is available. In terms of finding PRDs, the structured-based method in PrePPI_Struct applies a filtering criterion to ensure that the candidate PRD aligns well structurally to the template PRD at the binding interface, which is not accounted for by the sequence-based Pfam definition in PrePPI_PRD/motif.

As in the original PrePPI, combining sources of evidence that on their own provide only weak clues has a major impact on overall performance. For example, the consensus sequence patterns used in the PrePPI_PRD/motif approach can be promiscuous as can the use of sequence similarity in PrePPI_Struct, potentially leading to reduced prediction specificity. This can be especially problematic for interactions between candidate PRDs and motifs that interact via similar interfaces. For example, for the structural modeling component in PrePPI_Struct, it is possible that modeled interfaces for many different pairs of proteins share the same sets of clues if they are derived from the same template structure. In this case, prediction specificity would come from non-structural evidence. Moreover, prediction coverage based on the individual sources of evidence can be low as shown in Results, which highlights the importance of combining different sources of orthogonal information implicit in the Bayesian approach.

It is widely appreciated that HT approaches including yeast-two hybrid and tandem affinity purification have limitations in detecting PPIs mediated by protein-peptide interfaces. Apart from issues such as their transient nature and high K_d,_ they frequently depend on cellular conditions, many of which will never be sampled in an HT experiment [16, 19], potentially resulting in very high false negative rates. Indeed, high-throughput screens focusing on individual PRDs often identify a surprisingly large number of binding partners [19]. Furthermore, it has recently been suggested [17] that the number of putative peptide motifs in the human proteome to be more than a million. The number was based on estimating the extent of disordered regions in the human proteome and the tendencies of these regions to be involved in binding. Motifs that undergo post-translational modification were also included in the estimate, based on their prevalence among a set of well-studied proteins [17]. Although there are certainly false positives in computational predictions, these issues highlight the importance of developing methods such as that described here that can be applied on a genome wide scale and are insensitive to such experimental difficulties. The large number of predictions we make provide hypotheses that can be further refined and tested by more in-depth experimental/computational studies. In addition, the integrative nature of our framework should also help provide the biological context for predicted interactions, further contributing to our understanding of this still largely unexplored portion of the human interactome.

## Methods

### Human proteins

A total of 20,318 unique human protein sequences were downloaded from UniProt [39] and constituted the human proteome in this study.

### Identification of domains and motifs for PrePPI_PRD/motif

As of January 2014, a total of 203 ELM classes of motifs that shared similar sequence features and targeted by the same kind of domain were annotated in the ELM database. For each class, a consensus pattern for the motifs and the name of the Pfam family for the interacting PRD were retrieved from the database. Hidden Markov Models (HMMs) for each Pfam family were downloaded from the Pfam [40] website, and the hmmscan utility from the HMMER suite [41] was used to identify domains within each human protein using default cutoffs defined in the downloaded HMM files. Candidate motifs satisfying the consensus pattern were identified using an in-house Perl script.

### Identification of domains and motifs for PrePPI_Struct

We obtained domain-motif structures from the PepX database [34] (multimers interacting with a single peptide were excluded). A PRD in PepX was used as a template to model a domain-motif interaction for a given human protein if it is structurally similar to the model for that protein as defined by a protein structural distance (PSD) less than 0.65 calculated with the program Ska [42]. An additional requirement is that in the structural alignment at least 75% of interfacial residues for the template PRD must align to surface residues on the structural model for the protein. Interfacial residues for the template PRD were defined as those with at least one atom located within 4.5 Å of the template peptide motif in the complex structure. Surface residues for the structural models of human proteins were identified using the program SURFace [43], with an accessible surface area cutoff of 10 Å^2^. PSD scores between candidate domains and the template PRDs were grouped into two bins, [0–0.3] and [0.3–0.65], for the Bayesian classifier.

For a peptide motif in a given template that is x residues long, new potential binding motifs were identified by scanning a x-residue window across the whole human proteome. A sequence similarity score between the sliding window and the template motif was calculated using the BLOSUM62 scoring matrix [37]. Sequence motifs whose scores ranked among the top 0.05% among all such sliding windows were kept as candidate motifs. A cutoff based on percentage but not absolute BLOSUM62 scores enables comparison of motifs across different templates, which can vary greatly in length. For the Bayesian classifier, sequence similarity scores between candidate motifs and the template motifs were grouped into 4 bins: (1) scores within the top 0.0001%, (2) scores between the top 0.0001% and the top 0.001%, (3) scores between the top 0.001% and the top 0.01%, and (4) scores between the top 0.01% and the top 0.05%.

### Prediction of disorder

The program IUPred [44] was used to predict if a candidate motif is likely located in a disordered region. A disorder score (ranging from 0 to 1) for each individual residue in the human proteome was obtained by running IUPred on all human protein sequences. For each motif, a score was then obtained by averaging the disorder score for each individual residue in the motif. For the Bayesian scoring, a binary classification of candidate motifs was used: a candidate motif is disordered if the averaged score is larger than 0.5, which is the cutoff recommended by IUPred.

### Calculation of sequence conservation scores

The program GOPHER [45] was used to search for orthologs among the UniProt database for every human protein. Only orthologs belonging to species of the subphylum vertebrata were considered, as including orthologs from a larger range of species (e.g. metazoan) does not significantly impact performance. A multiple sequence alignment of the orthologs was then generated using the program Muscle [46]. A conservation score for each residue in the human protein was calculated as the information content for the corresponding column in the alignment. The score was multiplied by the percentage of non-gap residues in the column. A residue was determined to be conserved locally if its conservation score was higher than the average of such scores for its surrounding residues [47] (up to 31-residue upstream and downstream). For the Bayesian scoring, a binary classification of candidate motifs was used: a motif was classified as locally conserved if all residues in the candidate motif were locally conserved.

### Training set and the naïve Bayes classifier

A naïve Bayes classifier was used to integrate different sources of evidence into a likelihood ratio (LR) for an interaction between two proteins. The datasets for training the classifier consist of a true positive set that includes 7,409 interactions compiled from a set of 5 databases [48–52] and supported by at least two publications, and a true negative set that contains 206,361,949 interactions not supported by any publication [13]. While one can assume that a non-reported interaction is likely to be non-interacting, the negative set will necessarily contain undiscovered true interactions which are just the ones we would like to detect. The reported FPR at a given LR (which assumes every prediction in the true negative set is wrong) can therefore be viewed as an upper bound on the true value. As constructing a reliable set of non-interacting proteins remains difficult, we proceeded with this simple definition.

For Fig 2, in order to compare to iELM, we used a small set of 1.6 million pairs of proteins randomly sampled from the larger negative set, for which iELM scores were available. Results from the larger set were shown in Fig 3 (performance for both sets was nearly identical). For each property (referred to as a “clue”), *c*_*i*_, of an interaction between protein x and y, the conditional probability that one will observe the clue given that the interaction is in the true positive set or the true negative set is calculated as *P(c*_*i*_ *|I*_*xy*,*TP*_ *)* and *P(c*_*i*_ *|I*_*xy*,*TN*_ *)*. The probability *P(c*_*i*_ *|I*_*xy*,*TP*_ *)* is calculated as *P(c*_*i*_ *|I*_*xy*,*TP*_ *) = n/N*_*TP*_, where *n* is simply the number of interactions in the true positive set with the clue *c*_*i*_, and *N*_*TP*_ is the total number of interactions in the true positive set. *P(c*_*i*_ *|I*_*xy*,*TN*_ *)* is calculated analogously for the true negative set. A LR value can be calculated by dividing these two probabilities, *LR(c*_*i*_ *) = P(c*_*i*_ *|I*_*xy*,*TP*_ *) / P(c*_*i*_ *|I*_*xy*,*TN*_ *)*, to reflect how strongly the clue *c*_*i*_ is indicative of a true interaction.

For the PRD/motif method based on the ELM database, a total of four clues were used for the domain-motif component: a) whether a domain-motif match from the same ELM class is present (*LR(match)*); b) the identity of the matching ELM class (*LR(class)*); c) whether the motif is located in a predicted disordered region (*LR(diso)*); d) whether the motif is conserved locally in sequence relative to its surrounding regions (*LR(consv)*). The latter three clues can be assumed to be independent of one another, but they all depend on the first clue being true. Their LR values were therefore normalized by the LR for the first clue, and the final LR for the domain-motif interface is therefore:

LR(DMI)=LR(match)⋅(LR(class)/LR(match))⋅(LR(diso)/LR(match))⋅(LR(consv)/LR(match))

For the Struct method based on the PepX database, a total of five clues were used for the domain-motif component: a) whether a domain-motif match from the same template structure is present (*LR(match)*); b) The PSD score between the candidate domain and the PRD component in the template (*LR(PSD)*); c) the sequence similarity score between the candidate motif and the motif component in the template (*LR(SIM)*); d) whether the motif is located in a predicted disordered region (*LR(diso)*); e) whether the motif is conserved locally in sequence relative to its surrounding regions (*LR(consv)*). As above, LRs for the latter four clues were normalized by *LR(match)* and the final LR for the domain-motif interface is:

$$\begin{array}{l} LR(DMI)=\\ LR(match)\cdot (LR(PSD)/LR(match))\cdot (LR(SIM)/LR(match))\cdot (LR(diso)/LR(match))\cdot (LR(consv)/LR(match)) \end{array}$$

The LR for the domain-motif interface was then multiplied with LRs for non-structural evidence to obtain a final LR for the interaction. The LR values used in this study are provided as a supplemental table (S1 Table). LR scores for non-structural evidence based on co-expression, similarity in gene ontology, and similarity in phylogentic profiling calculated for the original PrePPI were used in this study [13].

Precision-recall curves were generated using the program AUCCalculator[53].

### Evaluating iELM

The iELM scores for the positive set and the randomly generated smaller negative set were kindly provided by Weatheritt et al. Incremental cutoffs of raw iELM scores were used to calculate the TPR and FPRs. If iELM makes multiple PRD/motif predictions for a single pair of protein, only the prediction with the highest score was considered in testing.

### Availability

Predictions have been incorporated into the PrePPI database and can be downloaded for individual query proteins (https://honiglab.c2b2.columbia.edu/PrePPI/). New predictions are also provided as a supplement (S2 Table).

## Supporting Information

S1 Fig Precision-recall curves to evaluate prediction performances. The same data sets as in Fig 2 were used, but performances were shown as precision vs. recall instead of true positive rate vs. false positive rate.
(TIF)

S2 Fig Improving PrePPI by adding domain-motif prediction methods. Same as Fig 3, but performances are evaluated on a smaller negative set as described in text.
(TIF)

S1 Table LR values for clues used in (A) PRD/motif and (B) Struct. (XLSX)

S2 Table List of new predictions. (XLSX)
