# Supplementary figures and images for: Predicting Peptide-Mediated Interactions on a Genome-Wide Scale
Source: PLoS Comput Biol. 2015 May 4;11(5):e1004248. doi: 10.1371/journal.pcbi.1004248 (PMC4418708; doi:10.1371/journal.pcbi.1004248)

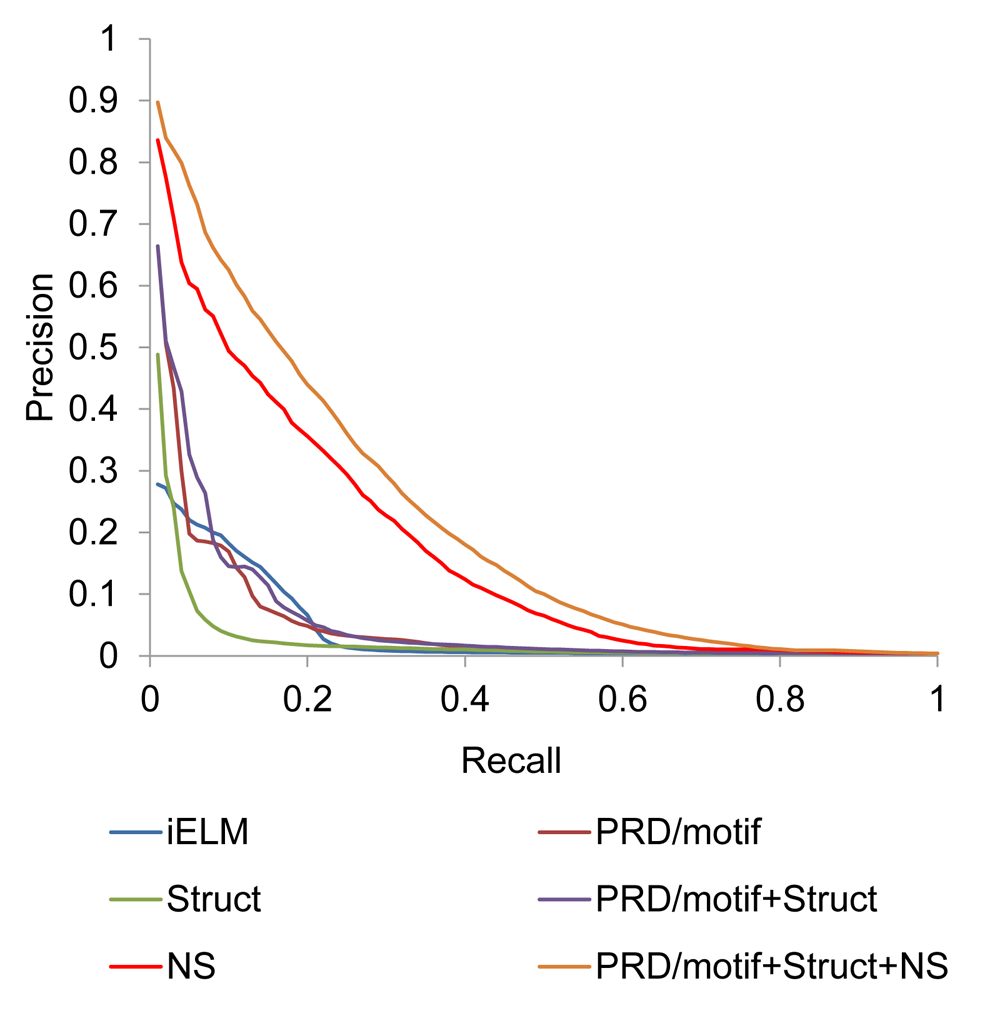

Supplement: S1 Fig — The same data sets as in Fig 2 were used, but performances were shown as precision vs. recall instead of true positive rate vs. false positive rate. (TIF) [file pcbi.1004248.s001.tif]

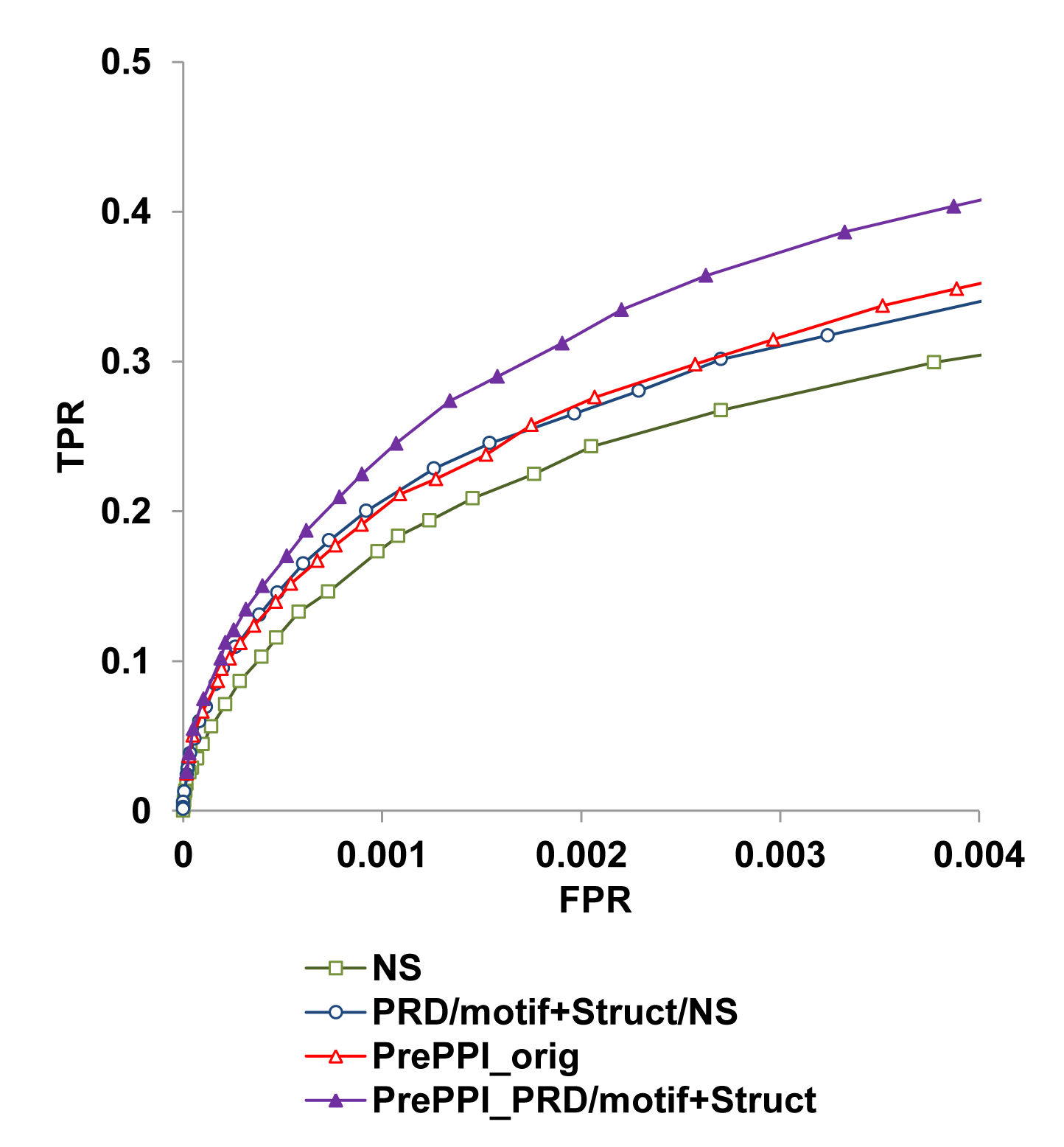

Supplement: S2 Fig — Same as Fig 3, but performances are evaluated on a smaller negative set as described in text. (TIF) [file pcbi.1004248.s002.tif]
